# Progressive Medicine, Vol. II, June, 1902

**Published:** 1902-08

**Authors:** 


### Books and Magazines.

PROGRESSIVE MEDICINE, VOL. II, JUNE, 1902. A Quarterly Digest of Advances, Discoveries and Improvements in the Medical and Surgical Sciences. Edited by Hobart Amory Hare, M. D., Professor of Therapeutics and Materia Medica in the Jefferson Medical College of Philadelphia. Octavo, handsomely bound in cloth, 440 pages, 28 illustrations. Per volume, $2.50, by express to any address. Per annum, in four cloth-bound volumes, $10.00. Lea Brothers & Co., Publishers, Philadelphia and New York.

This number of *Progressive Medicine* contains some unusually interesting matter.

Dr. William B. Coley devotes 150 well-written pages to the surgery of the abdomen, including hernia. Many conditions hitherto not looked upon as surgical affections are considered in this section. The writer's descriptions of new operations, and of the recent modifications of old ones, are more than mere outlines. They are sufficiently long and complete to be of practical value to the surgeon.

Dr. John G. Clark's section reviews the recent advances made in gynecology. All of the late devices for the treatment of the diseases of women, as well as gynecological technique and pathology, are fully covered in this article.

Dr. Alfred Stengel handles well an unusually difficult subject in his section on the diseases of the blood and ductless glands, the hemorrhagic and metabolic diseases.

The remainder of the volume is taken up by Dr. Edward Jackson on ophthalmology. R.

